# Clinical and therapeutic aspects of the alcohol addiction phenomenon in elderly women

**DOI:** 10.1192/j.eurpsy.2021.1496

**Published:** 2021-08-13

**Authors:** I. Sosin, G. Mysko, O. Honcharova, O. Misna, O. Minko

**Affiliations:** 1 Narcology Department, Kharkiv Medical Academy of Postgraduate Education, Kharkiv, Ukraine; 2 Department Of Urgent Psychiatry And Narcology, INPN NAMSU, Kharkiv, Ukraine

**Keywords:** Alcohol addiction, women, Treatment

## Abstract

**Introduction:**

Age-related features of alcohol addiction in elderly women (AAEW) have not been studied properly. The WHO classifies 60-75 years as elderly age (‘early old age’), when morphological and physiological functions of all organs and systems fade away, causing severe post-intoxication and withdrawal disorders, giving organic tint to alcohol dependence clinical picture, and rapid onset of alcoholic mental degradation of personality.

**Objectives:**

To study specific clinical, diagnostic and pathophysiological basis of alcohol dependence in aged women for innovative approaches to AAEW treatment.

**Methods:**

Clinical and medical history questioning, international tests and scales to identify alcohol dependence and complications in elderly women. Follow-up monitoring of basic biochemical, clinical, laboratory and electrophysiological findings at treatment runtime.

**Results:**

Multifactorial study and specific gender features in AAEW development allowed to identify abundant dual comorbidity, prevalence, high degree of affective disorders (depression, anxiety, dysphoria) combined with various somatic conditions and diencephalic symptoms in this alcoholic disease pattern. New treatment modality for alcohol dependence in elderly women was proposed and tested; along with classical detoxification and symptomatic therapy, the patients received anxiolytic agent (serotonin receptor stimulator) Buspirone SANDOZ, 5 mg 3 times a day, followed by individually corrected effective dose. The drug stopped anxiety, balanced the mood, causing no addiction. Buspirone was combined with bromine and sodium sulfate transcerebral electrophoresis № 5 and selective psychotherapy.

**Conclusions:**

The proposed integrated therapy for AAEW was proven to be effective by statistical reliability and patient-specific clinical illustrations.

